# Blastocoele expansion: an important parameter for predicting clinical success pregnancy after frozen-warmed blastocysts transfer

**DOI:** 10.1186/s12958-019-0454-2

**Published:** 2019-01-23

**Authors:** Jing Zhao, Yi Yan, Xi Huang, Lunquan Sun, Yanping Li

**Affiliations:** 1 Reproductive Medicine Center, Xiangya Hospital, Central South University, 87 Xiangya Road, Changsha City, Hunan Province People’s Republic of China; 2 Center for Molecular Medicine, Xiangya Hospital, Central South University, Hunan, People’s Republic of China

**Keywords:** Blastocoele expansion, Inner cell mass, Trophectoderm, Blastocyst morphology, Frozen–warmed embryo transfer

## Abstract

**Objective:**

To assess the predictive value of each individual morphological parameter: blastocoele expansion degree, inner cell mass (ICM), and trophectoderm (TE) grades on the clinical pregnancy outcome in frozen–warmed embryo transfer (FET) cycles.

**Methods:**

This is a retrospective cohort study, including 1154 FET cycles receiving vitrified-warmed one or two blastocysts transfer from August 2011 through to May 2018. The correlation between blastocyst morphology parameters and clinical outcome after FET was assessed.

**Results:**

In the subgroup analysis based on clinical pregnancy, the patients who achieved clinical pregnancy had a significantly higher degree of blastocyst expansion (3.69 ± 0.68 vs. 3.53 ± 0.78, *P* = 0.000) and had a thicker endometrium (9.65 ± 1.63 vs. 9.28 ± 1.64) compared with those with non-clinical pregnancy. The logistic regression analysis showed that among the three blastocyst morphology parameters, only the blastocoele expansion degree was significantly correlated with the clinical pregnancy outcome and had ability to predict the outcome after FET cycles with one or two vitrified-warmed blastocysts transferred. Both ICM and TE stages were not associated with pregnancy outcomes.

**Conclusions:**

The blastocoele expansion degree may be essential for successful pregnancy and should be given priority when selecting frozen blastocyst for transfer.

**Electronic supplementary material:**

The online version of this article (10.1186/s12958-019-0454-2) contains supplementary material, which is available to authorized users.

## Introduction

With the successful introduction of embryo vitrification, vitrified-warmed embryo transfer cycles, which preserve excess embryos derived from IVF or ICSI treatment cycles and transfer later, are a vital part of assisted reproductive technology (ART). Compared with cleavage embryo, blastocyst culture as a tool for selecting embryo has improved clinical pregnancy outcomes [1–3]. Besides, blastocyst transfer was regarded to enhance the synchrony between embryonic development and endometrium [4]. So, in the clinical practice, blastocyst vitrification and frozen – warmed embryo transfer (FET) in the following cycles is suggested when there have surplus embryos.

Unlike the intricate ovarian stimulation protocols used to stimulate multiple follicular developments, the FET protocols are simpler, and its purpose is limited to adequate preparation of the endometrium and selection good-quality embryos/blastocysts. In 2013, one meta-analysis showed that all current endometrial preparation methods appear to be equally successful in terms of ongoing pregnancy rate [5]. Therefore, selecting good-quality embryos with optimal implantation potential is critical to ensure that each infertile couple has the maximum likelihood of getting pregnant after ART. However, there is poorly consensus on the blastocyst selection.

At present, morphological evaluation has been widely used to predict embryo vitality as a relatively simple and non-invasive method. Several blastocyst-grading systems have been proposed to assess blastocyst quality. The blastocyst grading system proposed by Gardner and Schoolcraft [6] was based on blastocyst morphology parameters and was kept basically unchanged. Some studies have reported that trophectoderm (TE) grade has a stronger predictive power than the ICM grade and the blastocyst expansion degree in estimation of outcome after blastocyst transfer [7–10] while some studies showed that the ICM grade was more important [11, 12]. However, some researches indicated that the clinical outcomes after single blastocyst transfer could be predicted by blastocoele expansion degree [9, 13, 14]. And recently, Sun YP firstly confirmed that the blastocoele expansion and re-expansion degree had a stronger ability to predict live birth than ICM or TE grade in fresh and vitrified/ warmed single blastocyst transfer cycles [5].

This relationship among each parameter of the blastocyst morphology and outcomes of ART is inconsistent and is not fully understood. In addition, as far as we know, the correlation between blastocyst parameters and outcomes after transferring non-selective single or two blastocysts in FET cycles has not been examined.

So, the aim of the present study was to assess the prognostic value of each individual morphological parameter (blastocoele expansion degree, ICM, and TE grade) on the clinical pregnancy outcome in FET cycles.

## Materials and methods

### Study design

This study was approved by the Ethical Committee of Xiangya Hospital, Central South University, and was performed in the light of the Declaration of Helsinki. This study retrospectively analyzed infertile couples who received frozen-warmed blastocyst transfer at the Reproductive Medical Center of the Xiangya Hospital from August 2011 through to May 2018. The inclusion criteria: 1) frozen-warmed blastocyst transfer cycles with one or two blastocysts transferred; 2) for women who received two blastocysts transferred, cycles in which non or two blastocysts implanted were included. The main exclusion criteria: 1) preimplantation genetic diagnosis/screening (PGD/PGS) cycles; 2) women with intrauterine adhesion (IUA); 3) fresh blastocyst transfer cycles.

### Ovarian stimulation protocol

All the vitrified/warmed blastocysts transferred during the later cycles were acquired during ovarian stimulation cycles as described previously [15–18]. The personalized individual ovarian stimulation protocol was given basing on the age, infertility factor, AMH, and antral follicular count (AFC). When the serum estrogen < 50 pg/ml, and diameter of the largest follicle < 10 mm, ovarian stimulation initiated. One hundred fifty to Three hundred IU recombinant FSH and/or human menopausal gonadotrophin were used to promote follicle development. Follicle was triggered with 5000–10,000 IU of hCG when at least two follicles > 18 mm. Oocytes were picked up 36 h after hCG injection, and IVF or ICSI was conducted according to the practice protocol.

### Blastocyst vitrification and warming

If there were surplus embryos after transfer in fresh cycles, these embryos were suggested to be cultured to form blastocyst. According to the methods reported by Lane et al. [19] and Mukaida et al. [20, 21], blastocysts were vitrified and warmed. Embryologists artificially shrink the blastocysts with a laser pulse prior to vitrification. Blastocysts were firstly transferred to an equilibration solution composed of 7.5% DMSO and 7.5% ethyleneglycol for 8–10 min, and then were transferred to a vitrification solution containing 15% DMSO + 15% ethylene glycol + 0.5 M sucrose for 1 min. Subsequently, each blastocyst was placed into a cryoloop, plunged into liquid nitrogen, and then covered with a cryotube and stored in liquid nitrogen.

To warm, the blastocyst loaded in the cryoloop was immersed in the first thawing solution with 1.0 M sucrose at room temperature for 2 min, and then transferred to the second thawing solution containing 0.5 M sucrose for 3 min. At last, blastocysts were washed in the wash solution, at 37 °C for 10 min. After warming up, the blastocysts were cultured in culture medium for 3 h.

In light of Gardner and Schoolcraft’s blastocyst grading system, vitrified/ warmed blastocysts were graded 2 h after thawing of blastocyst [6]. Blastocysts were graded by two embryologists who have more the 5 years of work experience, aiming to decrease subjective errors. Blastocysts with >3BB grade were deemed to be viable and were transferred.

### Frozen-warmed blastocyst transfer cycle

In the natural cycle FET (NC-FET), we monitored the development of the dominant follicle and endometrium from the 10th day of menstrual cycle by regular vaginal ultrasound, combined with urine LH, serum LH, E_2_ and progesterone measurements until ovulation. One or two warmed blastocysts were transferred 5 days after ovulation.

In the hormone replacement treatment FET cycles (HRT-FET), women received escalating doses of estradiol valerate (4 mg daily since cycle day 3 for 6 days, 6 mg daily for 6 days). Endometrial thickness and pattern were monitored by vaginal ultrasound, and when the endometrial thickness was ≥7 mm, progesterone (60 mg daily) was administrated. One or two blastocysts were transferred 5 days after progesterone administration.

In the ovulation-induction cycle FET (OI-FET), drugs such as letrozole were given to promote the development of the ovarian follicles from the 3rd day of cycle to the 7th day. Endometrium and follicle were monitored by vaginal ultrasound from the 10th day of the cycle, and 37.5 IU - 75 IU HMG was administrated if necessary. When the largest follicle exceeded 18 mm, 10,000 IU of hCG was injected to trigger ovulation. One or two blastocysts were transferred 5 days after ovulation.

As for the GnRH-a + HRT FET protocol, GnRH-a 3.75 mg was injected on the 2nd or the 3th day of the menstrual cycle. After 14 days GnRH-a injection, the estradiol valerate was administrated according to the HRT-FET protocol. Estradiol valerate was commenced orally with a step-up from 4 mg daily for 6 days, to 6 mg daily for 6 days. If the endometrial thickness was ≥7 mm, progesterone (60 mg daily) was administrated.

In all FET cycles, one or two cryopreserved blastocysts were warmed and transferred. The primary outcome was clinical pregnancy, and the secondary observation index was biochemical pregnancy. Clinical pregnancy was confirmed by ultrasound visualization of a gestational sac 4–5 weeks after blastocyst transfer. Biochemical pregnancy was determined with a serum hCG > 10 IU/L 12–14 days after blastocyst transfer. Luteal support would last for 1 month at least after clinical pregnancy confirmation.

### Statistical analysis

All data analyses were conducted with SPSS software version 23.0 (IBM, New York, USA). Continuous data were showed as the mean ± standard deviation (SD), and subjected to a Student’s t-test. Categorical data were described as a frequency and percentages and analyzed using a Chi-square test. The relationship between pregnancy outcomes and morphological variables were analyzed by multivariate logistic regression analysis. *P* < 0.05 was set as statistically significant.

## Results

In total, 1154 frozen-warmed blastocysts transfer cycles were included, involving 725 single-blastocyst transfer cycles and 429 two-blastocysts transfer cycles. In total, 1583 viable blastocysts were transferred, and 568 blastocysts implanted. The implantation rate was 35.88% (568/1583) and the clinical pregnancy rate was 37.6% (434/1154). The majority of blastocysts were of expansion grade 4 (57.30%) and 3 (31.65%). Characteristics of patients and blastocyst morphology were summarized in Table 1.

**Table 1:** Characteristic of patients and blastocyst morphology (*N* = 1154)

Characteristic	Mean ± SD / N / %
Female age(years)	31.39 ± 5.48
No. of early cycles	2.65 ± 1.06
Duration of infertility (years)	5.68 ± 3.13
Endometrial thickness (mm)	9.42 ± 1.65
Expansion of blastocyst	3.59 ± 0.75
degree 1	21(1.33%)
degree 2	87(5.50%)
degree 3	501(31.65%)
degree 4	907(57.30%)
degree 5	52(3.28%)
degree 6	15(0.95%)
Cycles of clinical pregnancy	434(37.6%)
Cycles of non-pregnancy	720(62.4%)
Cycles with single blastocyst transfer	725(62.8%)
Cycles with two blastocysts transfer	429(37.2%)
Total No. of blastocysts transferred	1583
Cycles with one embryo sac	300
Cycles with two embryo sacs	134
Total No. of blastocyst implanted	568
Implantation rate	35.88%
Type of infertility	
Primary infertility	518(44.9%)
Secondary infertility	636(55.1%)
Protocol for endometrial preparation	
GnRH-a + HRT-FET	82(7.1%)
HRT-FET	627(54.3%)
OI-FET	51(4.4%)
NC-FET	394(34.1)

Categorical variable is present as n (%), and continuous variable is present as Mean ± SD. *NC* Natural cycle, *HRT* Hormone replacement treatment, *OI* ovulation-induction

**Table 2:** Characteristics of patient and blastocyst morphology divided by clinical pregnancy

Variable	Clinical pregnancy (*n* = 434)	Non-clinical pregnancy (*n* = 720)	*P*-value
Female age(y)	30.68 ± 5.25	31.39 ± 5.70	0.034
Duration of infertility(y)	5.77 ± 2.94	5.63 ± 3.24	0.731
Type of infertility			
Primary infertility	215(49.5%)	303(42.1%)	
Secondary infertility	219(50.5%)	417(57.9%)	0.140
No. of early cycles	2.66 ± 1.04	2.64 ± 1.07	0.794
Protocol of FET			
NC-FET	154(35.5%)	240(33.3%)	
HRT-FET	220(50.7%)	407(56.5%)	
OI-FET	20(4.6%)	31(4.3%)	
GnRH-a + HRT-FET	40(9.2%)	42(5.8%)	0.090
No. of blastocyst transferred	1.34 ± 0.48	1.39 ± 0.49	0.121
Endometrial thickness (mm)	9.65 ± 1.63	9.28 ± 1.64	0.000
Endometrial pattern			
A	123(28.3%)	187(26.0%)	
B	280(64.5%)	479(66.5%)	
C	31(7.1%)	54(7.5%)	0.677
Blastocoele expansion	3.69 ± 0.68	3.53 ± 0.78	0.000
1	5(0.9%)^a,b,c,d^	16(1.6%) ^a,b,c,d^	
2	21(3.6%)^c,d^	66(6.6%) ^c,d^	
3	156(26.8%)^b,d^	345(34.5%) ^b,d^	
4	373(64.0%)^a^	534(53.4%) ^a^	
5	24(4.1%)^a,b,c,d^	28(2.8%) ^a,b,c,d^	
6	4(0.7%)^a,b,c,d^	11(1.1%) ^a,b,c,d^	
ICM grade			
A	83(14.2%)	127(12.7%)	
B	445(76.3%)	785(78.5%)	
C	55(9.4%)	88(8.8%)	0.594
TE grade			
A	96(16.5%)	148(14.8%)	
B	416(71.4%)	694(69.4%)	
C	71(12.2%)	158(15.8%)	0.122

Variables were present as mean ± SD, % or n

*ICM* inner cell mass, *TE* trophectoderm

For blastocoele expansion evaluation, groups (in the same columns) with the same letters have no significantly difference

According to clinical pregnancy outcome, 1154 frozen blastocyst transfer cycles were divided into two groups: clinical pregnancy group (*n* = 434) and non-clinical pregnancy group (*n* = 720). There were no significantly differences between the two groups concerning the duration of infertility, No. of early cycles, protocol for endometrial preparation, the pattern of endometrium, No. of blastocyst transferred, distribution of ICM and TE. The patients who achieved clinical pregnancy were younger (30.68 ± 5.25 vs. 31.39 ± 5.70, *P* = 0.034), and had a significantly thicker endometrium (9.65 ± 1.63 vs. 9.28 ± 1.64, *P* = 0.000) and a significantly higher degree of blastocyst expansion (3.69 ± 0.68 vs. 3.53 ± 0.78, *P* = 0.000) compared with those with non-clinical pregnancy. In the three blastocyst morphology parameters, the degree of blastocoele expansion was the most relevant factor for clinical pregnancy (Table 2).

According to biochemical pregnancy, 1154 cycles were divided into biochemical pregnancy group (*n* = 599) and non-biochemical pregnancy group (*n* = 555). Like the results of clinical pregnancy evaluation, there were no significantly differences in the duration of infertility, the type of infertility, number of early cycles, protocol of FET, No. of blastocyst transferred, endometrial pattern, ICM grade between the two groups (*P* > 0.05). Women in biochemical pregnancy group were younger than those without biochemical pregnancy (31.04 ± 5.31 vs. 31.80 ± 5.77, *P* = 0.019), and had a significantly thicker endometrium compared with women without biochemical pregnancy (9.52 ± 1.61 vs. 9.31 ± 1.68, *P* = 0.032). When the blastocyst parameters were evaluated, the mean EH stage of blastocyst transferred was higher in the women with biochemical pregnancy than those without biochemical pregnancy (3.66 ± 0.69 vs. 3.51 ± 0.79, *P* = 0.000). The biochemical pregnancy group had a larger proportion of blastocyst with TE grade B (71.9%) and smaller proportion of TE grade C (12.4%) compared with women without biochemical pregnancy (Additional file 1 Table S1).

With logistic regression analysis, we assessed the correlation between clinical pregnancy/biochemical pregnancy and blastocyst morphology parameters and cycle characteristics (For biochemical pregnancy, see Additional file 2: Table S2). For clinical pregnancy, the endometrial thickness and the blastocoele expansion degree were significantly associated. Although the difference in the endometrial thickness was significant, the absolute difference in endometrial thickness between groups was less than 1 mm and was considered to be of no clinical significance. The clinical pregnancy rate increased from 23.8% with blastocoele expansion degree 1 to 46.2% with blastocoele expansion degree 5. The other characteristics including female age, type of infertility, No. of early cycles, protocol of FET, endometrial pattern, No. of blastocyst transferred, ICM grade and TE grade, were not significantly relevant to the clinical pregnancy in this sample (Table 3). The clinical pregnancy rates of subgroups with different blastocoele expansion degree (degree 1–6) were 23.8, 24.1, 31.1, 41.1, 46.2 and 26.7%, respectively, and these values were obviously different (*P* = 0.000) (Additional file 3 Table S3). For biochemical pregnancy, besides the endometrial thickness and the blastocoele expansion degree, the blastocyst TE was associated with the outcome (*P* = 0.035). The biochemical pregnancy rates were 33.3, 40.2, 46.7, 56.7, 53.8, and 46.7% in the subgroups with blastocoele expansion degree 1–6, respectively, and there were significant differences between subgroups (*P* = 0.004) (Additional file 3 Table S3). When the blastocyst TE grade was evaluated, we found the TE grade B have the highest biochemical pregnancy rate (53.4%), followed by subgroup with grade A (53.3%), and grade C (44.5%), and there was significantly difference between subgroup with TE grade B and C (*P* = 0.046) (Additional file 3 Table S3).

**Table 3:** Logistic model for predicting clinical pregnancy

Variable	B	S.E.	Wald	df	Sig.	Exp(B)	95% CI for Exp(B)	
							Lower	Upper
Age	−0.021	0.012	2.963	1	0.085	0.979	0.957	1.003
Type of infertility	−0.242	0.128	3.586	1	0.058	0.785	0.611	1.009
No. of early cycles	0.020	0.059	0.119	1	0.731	1.021	0.908	1.147
Protocol of FET	/	/	5.072	3	0.167	/	/	/
Protocol of FET(1)	0.481	0.252	3.654	1	0.056	1.618	0.988	2.651
Protocol of FET(2)	−0.063	0.140	0.203	1	0.652	0.939	0.713	1.236
Protocol of FET(3)	0.073	0.317	0.053	1	0.817	1.076	0.578	2.004
Endometrial thickness	0.134	0.039	11.904	1	0.001	1.143	1.059	1.233
Endometrial pattern	/	/	0.683	2	0.711	/	/	/
Endometrial pattern(1)	0.152	0.261	0.340	1	0.560	1.164	0.698	1.942
Endometrial pattern(2)	0.043	0.244	0.031	1	0.859	1.044	0.648	1.683
No. of blastocyst transferred	−0.189	0.132	2.040	1	0.153	0.828	0.639	1.073
Blastocoele expansion	0.262	0.091	8.253	1	0.004	1.300	1.087	1.555
ICM grade	/	/	0.315	2	0.854	/	/	/
ICM grade(1)	0.158	0.300	0.279	1	0.597	1.172	0.651	2.108
ICM grade(2)	0.134	0.253	0.282	1	0.595	1.144	0.697	1.878
TE grade	/	/	2.682	2	0.262	/	/	/
TE grade(1)	0.324	0.246	1.733	1	0.188	1.383	0.853	2.242
TE grade(2)	0.315	0.195	2.608	1	0.106	1.371	0.935	2.011
Constant	−2.165	0.837	6.682	1	0.010	0.115	/	/

The study assessed the clinical pregnancy and biochemical pregnancy by combined blastocyst morphology. The results showed the clinical pregnancy and biochemical pregnancy rates were associated with the blastocoele expansion degree when they have the same ICM and TE grade, and transferring blastocyst with blastocoele expansion degree 4 and 5 had achieved a better clinical outcome. Whereas with the same blastocoele expansion degree, there was no significantly difference in clinical pregnancy rate and biochemical pregnancy rate between subgroups with different combined ICM grade and TE grade (*P* > 0.05) (Additional file 4 Table S4). For blastocysts with expansion 3–5, TE grade had a more effect on the biochemical pregnancy with positive HCG, clinical pregnancy (Additional file 4 Table S4).

## Discussion

The present study showed that blastocoele expansion degree was significantly associated with the clinical pregnancy and biochemical pregnancy rates after one or two frozen blastocysts transfer. It has been reported that blastocoel expansion is important for clinical outcomes. A study by Goto et al. [11] investigated 1488 FET cycles with single blastocyst transfer and found that it was the blastocoele expansion degree rather than ICM/ TE that significantly affected pregnancy outcomes. Ahlstrom et al. [7] and Thompson et al. [9] reported that the blastocyst expansion degree was a significant and independent predictor of live birth rates of fresh single blastocyst transfer cycle. Recently, one study by Sun YP et al. [5] confirmed that the blastocoele expansion degree had a better predictive value than ICM or TE grade in predicting live birth after both fresh and vitrified/ warmed single blastocyst transfer cycles.

As stated in previous researches, blastocoele expansion stage has a lot of significant functions in the early stages of blastocyst implantation. These functions involve hatching from the zona pellucida, hCG-mediated signaling, adhesion and invasion of the endometrium, and dialogue with the mother [22–27], which are essential for a successful pregnancy as well as prevention of abortion. What have been stated above may explain our results that blastocoele expansion degree has more predictive information than TE and ICM stage.

Certainly, there have been many studies indicated the predictive value of TE or ICM stage. The possible explanations for such different conclusions may be as follows: Firstly, the distribution of different grades blastocysts is diverse between studies, which may explain the obvious inconsistency. For example, some studies evaluated the effect of blastocyst parameters on the clinical outcome after selective single blastocyst transfer in fresh cycles or in FET cycles. Most of blastocysts transferred have good or even excellent quality, and a very small part of blastocysts has C grade for ICM or TE. But, the data in the present study were derived from a retrospective clinical trial in which one or two blastocysts were transferred regardless of the quality of blastocyst, thus a number of blastocysts with blastocoele expansion degree 1–2 and ICM and TE with grade C were included. Secondly, in spite of the fact that these studies applied the same blastocyst grading system with written definitions and common training courses, the discursive concept of the ICM and TE morphology was an intrinsic defect in the blastocyst grading system proposed by Gardner and Schoolcraft [6], and subjective assessment was inevitable when grading the blastocyst.

In the present study, we found that majority of blastocysts had blastocoele expansion 3–5 and the grade of ICM and TE were highly correlated: ICM and TE A and B grade were more likely correlated with TE and ICM A and B grade, for example, AA, AB, BA, BB, or CC grades appeared more likely than other combinations (i.e. AC, CA grades). These observations showed interdependence between ICM and TE during the blastocyst developmental phase. As discussed by Ahlstrom et al. [7], the blastocoele expansion is relevant to the cell numbers and cohesive property of the TE, preventing outflow of blastocyst fluid and sodium ions. Therefore, a good TE grade may manifest that the blastocyst effectively pumps ions into the cavity and induces the permeability accumulation of intracellular water, leading to blastocoele expansion [7]. That is to say, by definition, functional TE cells are required to a fully expanded blastocyst, and this function may be more relevant to the molecular quality of TE cells than its number and cohesion. A better understanding of the correlation between these three morphological parameters helps to choose the best blastocyst for transfer.

Some studies suggested that blastocyst in earlier development stage and with lower blastocoele expansion degree have higher survival rate, implantation rate and live birth rates, which contradict to our findings [20, 28–31]. The method of vitrification used should be considered. Some studies have not used artificial shrinking of the blastocoele cavity to obtain higher-grade blastocysts. In these studies, the blastocoele fluid was regarded to be a barrier to dehydration and pervasion of cryoprotectant along with a negative impact on the blastocyst survival and implantation. Whereas, once blastocyst received artificial shrinkage, blastocysts with higher expansion degree owned the same or a higher survival rate, implantation rate and live birth rate compared to blastocysts with lower developmental degree [32–34]. Small blastomeres with larger surface area to volume ratio have been shown to be less sensitive to osmotic pressure and damage. The reason is that cryoprotectant penetrates into smaller cells faster [35]. Thus, higher expansion degree blastocysts with smaller balstomeres would most likely be more resistant to vitrification and high concentrations of cryoprotectants.

The strength of the present study was its large sample size of 1154 transfer cycles, and total 1583 warmed blastocysts transferred. Besides, different with previous studies which included good blastocyst, this study assessed all kinds of viable blastocysts >3CC and did not limit to good quality blastocyst (≥3BB). And our results found that ICM C grade or TE C grade blastocyst with higher blastocoele expansion degree also has optimistic clinical results. In addition, the present study firstly assessed FET cycles with single blastocyst or two blastocysts transfer, and cycles in which blastocysts transferred were all implanted or all not implanted were included. At present, selective single embryo/blastocyst transfer and blastocyst culture were not routine practices. When there still have surplus embryos after fresh embryo transfer, these embryos, including not top-quality embryos, were proposed to be cultured to blastocyst stage.

Certainly, this study has a few limitations. The first one was its retrospective design, and some bias was inevitable. Secondly, this study did not observe other clinical outcomes, such as ongoing pregnancy rate, abortion rate, and live birth rate. As the follow-up has not been completed in some cases by now, so it is hard to compare these outcomes. Besides, live birth was affected by too many factors except for blastocyst quality. So, the present included the biochemical pregnancy and clinical pregnancy, which were surely associated with blastocyst quality. Thirdly, the present study only evaluated the blastocoele expansion degree after embryo thawing, and did not evaluate the expansion degree before vitrification. Recent study showed that the degree of blastocyst expansion after vitrification/thawing was almost the same as that before vitrification [1]. In spite of these shortcomings, the present study analyzed sis the parameters of blastocysts with a valuable summary.

## Conclusion

The blastocyst expansion is a significant parameter for predicting clinical success pregnancy after frozen-thawed blastocysts transfer and should be given the priority when selecting frozen blastocyst for transfer.

## Additional files

Additional file 1: **Table S1.** Characteristics of patient and blastocyst morphology divided by biochemical pregnancy. (DOCX 88 kb)

Additional file 2: **Table S2.** Logistic model for predicting biochemical pregnancy. (DOCX 109 kb)

Additional file 3: **Table S3.**Comparison of clinical pregnancy rate and biochemical pregnancy rate between different subgroups according to blastocoele expansion degree and TE grade. (DOCX 70 kb)

Additional file 4: **Table S4.** Comparison of outcomes between different subgroups divided according by blastocyst ICM/TE grade with the same expansion degree. (DOCX 81 kb)

## Abbreviations

ART: assisted reproductive technology
FET: frozen-thawing embryo transfer
ICSI: intracytoplasmic sperm injection
IUA: intrauterine adhesion
IVF: in vitro fertilization
PGD/PGS: preimplantation genetic diagnosis/screening
