# Supplementary material for: Blastocoele expansion: an important parameter for predicting clinical success pregnancy after frozen-warmed blastocysts transfer
Source: Reprod Biol Endocrinol. 2019 Jan 23;17:15. doi: 10.1186/s12958-019-0454-2 (PMC6344998; doi:10.1186/s12958-019-0454-2)
Supplement: Supplementary file 1 — Table S1. Characteristics of patient and blastocyst morphology divided by biochemical pregnancy. (DOCX 88 kb) [file 12958_2019_454_MOESM1_ESM.docx]

Supplemental table 1 characteristics of patient and blastocyst morphology divided by biochemical pregnancy

| Variable | Biochemical  Pregnancy (n=599) | Non-biochemical pregnancy (n=555) | P-value |
| --- | --- | --- | --- |
| Female age (y) | 31.04±5.31 | 31.80±5.77 | 0.019 |
| Duration of infertility (y) | 5.65±3.08 | 5.78±3.20 | 0.647 |
| Type of infertility |  |  |  |
| Primary infertility | 293(48.9%) | 225(40.5%) |  |
| Secondary infertility | 306(51.1%) | 330(59.5%) | 0.052 |
| No. of early cycles | 2.68±1.09 | 2.62±1.03 | 0.339 |
| Protocol of FET |  |  |  |
| NC-FET | 205(34.2%) | 189(34.1%) |  |
| HRT-FET | 315(52.6%) | 312(56.2%) |  |
| OI-FET | 29(4.8%) | 22(4.0%) |  |
| GnRH-a+HRT-FET | 50(8.3%) | 32(5.8%) | 0.272 |
| No. of blastocyst transferred | 1.38±0.49 | 1.37±0.48 | 0.686 |
| Endometrial thickness (mm) | 9.52±1.61 | 9.31±1.68 | 0.032 |
| Endometrial pattern |  |  |  |
| A | 165(27.5%) | 145(26.1%) |  |
| B | 388(64.8%) | 371(66.8%) |  |
| C | 46(7.7%) | 39(7.0%) | 0.677 |
| Blastocoele expansion | 3.66±0.69 | 3.51±0.79 | 0.000 |
| 1 | 7(0.8%)^a,b,c,d^ | 14(1.8%) ^a,b,c,d^ |  |
| 2 | 35(4.2%)^c,d^ | 52(6.9%) ^c,d^ |  |
| 3 | 234(28.4%)^b,d^ | 267(35.2%) ^b,d^ |  |
| 4 | 514(62.3%)^a^ | 393(51.8%) ^a^ |  |
| 5 | 28(3.4%)^a,b,c,d^ | 24(3.2%) ^a,b,c,d^ |  |
| 6 | 7(0.8%)^a,b,c,d^ | 8(1.1%) ^a,b,c,d^ |  |
| ICM grade |  |  |  |
| A | 118(14.3%) | 92(12.1%) |  |
| B | 624(75.6%) | 606(79.9%) |  |
| C | 83(10.1%) | 60(7.9%) | 0.113 |
| TE grade |  |  |  |
| A | 130(15.8%)^a,b^ | 114(15.0%) ^a,b^ |  |
| B | 593(71.9%)^b^ | 517(68.2%) ^b^ |  |
| C | 102(12.4%)^a^ | 127(16.8%) ^a^ | 0.046 |

Variables were present as mean± SD, % or n.

ICM=inner cell mass; TE=trophectoderm.

For TE grade evaluation, sub-groups (in the same columns) with same letters have no significantly difference.
