# Supplementary material for: Blastocoele expansion: an important parameter for predicting clinical success pregnancy after frozen-warmed blastocysts transfer
Source: Reprod Biol Endocrinol. 2019 Jan 23;17:15. doi: 10.1186/s12958-019-0454-2 (PMC6344998; doi:10.1186/s12958-019-0454-2)
Supplement: Supplementary file 2 — Table S2. Logistic model for predicting biochemical pregnancy. (DOCX 109 kb) [file 12958_2019_454_MOESM2_ESM.docx]

Supplemental table 2 logistic model for predicting biochemical pregnancy

| Variable | B | S.E. | Wald | df | Sig. | Exp(B) | 95% CI for Exp(B) | |
| --- | --- | --- | --- | --- | --- | --- | --- | --- |
|  |  |  |  |  |  |  | Lower | Upper |
| Age | -0.019 | 0.011 | 2.953 | 1 | 0.086 | 0.981 | 0.959 | 1.003 |
| Type of infertility | -0.288 | 0.124 | 5.405 | 1 | 0.061 | 0.750 | 0.588 | 0.956 |
| No. of early cycles | 0.061 | 0.058 | 1.097 | 1 | 0.295 | 1.063 | 0.948 | 1.191 |
| Protocol of FET | / | / | 3.382 | 3 | 0.336 | / | / | / |
| Protocol of FET(1) | 0.412 | 0.255 | 2.610 | 1 | 0.106 | 1.510 | 0.916 | 2.489 |
| Protocol of FET(2) | -0.024 | 0.135 | 0.032 | 1 | 0.859 | 0.976 | 0.749 | 1.273 |
| Protocol of FET(3) | 0.152 | 0.311 | 0.240 | 1 | 0.624 | 1.164 | 0.633 | 2.140 |
| Endometrial thickness | 0.077 | 0.038 | 4.142 | 1 | 0.042 | 1.080 | 1.003 | 1.163 |
| Endometrial pattern | / | / | 0.570 | 2 | 0.752 | / | / | / |
| Endometrial pattern(1) | -0.078 | 0.252 | 0.096 | 1 | 0.756 | 0.925 | 0.565 | 1.515 |
| Endometrial pattern(2) | -0.149 | 0.235 | 0.402 | 1 | 0.526 | 0.862 | 0.544 | 1.365 |
| No. of blastocyst transferred | 0.083 | 0.127 | 0.425 | 1 | 0.514 | 1.086 | 0.847 | 1.392 |
| Blastocoele expansion | 0.261 | 0.088 | 8.894 | 1 | 0.003 | 1.299 | 1.094 | 1.542 |
| ICM grade | / | / | 0.400 | 2 | 0.819 | / | / | / |
| ICM grade(1) | -0.069 | 0.293 | 0.055 | 1 | 0.814 | 0.933 | 0.651 | 2.108 |
| ICM grade(2) | -0.136 | 0.247 | 0.301 | 1 | 0.583 | 0.873 | 0.697 | 1.878 |
| TE grade | / | / | 4.584 | 2 | 0.101 | / | / | / |
| TE grade(1) | 0.402 | 0.236 | 2.896 | 1 | 0.089 | 1.494 | 0.941 | 2.372 |
| TE grade(2) | 0.390 | 0.185 | 4.453 | 1 | 0.035 | 1.477 | 1.028 | 2.123 |
| Constant | -1.172 | 0.803 | 2.134 | 1 | 0.144 | 0.310 | / | / |
