# Supplementary material for: Blastocoele expansion: an important parameter for predicting clinical success pregnancy after frozen-warmed blastocysts transfer
Source: Reprod Biol Endocrinol. 2019 Jan 23;17:15. doi: 10.1186/s12958-019-0454-2 (PMC6344998; doi:10.1186/s12958-019-0454-2)
Supplement: Supplementary file 3 — Table S3.Comparison of clinical pregnancy rate and biochemical pregnancy rate between different subgroups according to blastocoele expansion degree and TE grade. (DOCX 70 kb) [file 12958_2019_454_MOESM3_ESM.docx]

Supplemental table 3 Comparison of clinical pregnancy rate and biochemical pregnancy rate between different subgroups according to blastocoele expansion degree and TE grade

|  | Clinical  pregnancy n(%) | Non-clinical pregnancy n(%) | P | Cramer V | Biochemical pregnancy n(%) | Non-biochemical pregnancy n(%) | P | Cramer V |
| --- | --- | --- | --- | --- | --- | --- | --- | --- |
| Blastocoele expansion degree |  |  |  |  |  |  |  |  |
| 1 | 5(23.8%) | 16(76.2%) |  |  | 7(33.3%) | 14(66.7%) |  |  |
| 2 | 21(24.1%) | 66(75.9%) |  |  | 35(40.2%) | 52(59.8%) |  |  |
| 3 | 156(31.1%) | 345(68.9%) |  |  | 234(46.7%) | 267(53.3%) |  |  |
| 4 | 373(41.1%) | 534(58.9%) |  |  | 514(56.7%) | 393(43.3%) |  |  |
| 5 | 24(46.2%) | 28(53.8%) |  |  | 28(53.8%) | 24(46.2%) |  |  |
| 6 | 4(26.7%) | 11(73.3%) | 0.00 | 0.00 | 7(46.7%) | 8(53.3%) | 0.001 | 0.001 |
| Blastocyst TE grade | / | / | / | / |  |  |  |  |
| A |  |  |  |  | 130(53.3%) | 114(46.7%) |  |  |
| B |  |  |  |  | 593(53.4%) | 517(46.6%) |  |  |
| C |  |  |  |  | 102(44.5%) | 127(55.5%) | 0.046 | 0.046 |
