# Supplementary material for: Blastocoele expansion: an important parameter for predicting clinical success pregnancy after frozen-warmed blastocysts transfer
Source: Reprod Biol Endocrinol. 2019 Jan 23;17:15. doi: 10.1186/s12958-019-0454-2 (PMC6344998; doi:10.1186/s12958-019-0454-2)
Supplement: Supplementary file 4 — Table S4. Comparison of outcomes between different subgroups divided according by blastocyst ICM/TE grade with the same expansion degree. (DOCX 81 kb) [file 12958_2019_454_MOESM4_ESM.docx]

| Supplemental table 4 comparison of outcomes between different subgroups divided according by blastocyst ICM/TE grade with the same expansion degree | | | | | |
| --- | --- | --- | --- | --- | --- |
| Blastocoele expansion |  | Clinical pregnancy | P | Biochemical pregnancy | P |
| 1 | AA (n=0) | / |  | / |  |
|  | AB (n=3) | 2(66.7%) |  | 2(66.7%) |  |
|  | AC (n=0) | / |  | / |  |
|  | BA (n=7) | 2(28.6%) |  | 2(28.6%) |  |
|  | BB (n=10) | 1(10%) |  | 3(30%) |  |
|  | BC (n=0) | / |  | / |  |
|  | CA (n=0) | / |  | / |  |
|  | CB (n=1) | 0(0) |  | 0(0) |  |
|  | CC (n=0) | / | 0.126 | / | 0.667 |
| 2 | AA (n=9) | 2(22.2%) |  | 5(55.6%) |  |
|  | AB (n=17) | 4(23.5%) |  | 8(47.1%) |  |
|  | AC (n=0) | / |  | / |  |
|  | BA (n=24) | 10(41.7%) |  | 13(54.2%) |  |
|  | BB (n=35) | 5(14.3%) |  | 9(25.7%) |  |
|  | BC (n=2) | 0 |  | 0 |  |
|  | CA (n=0) | / |  | / |  |
|  | CB (n=0) | / |  | / |  |
|  | CC (n=0) | / | 0.168 | / | 0.100 |
| 3 | AA (n=17) | 4(23.5%) |  | 6(35.3%) |  |
|  | AB (n=19) | 6(31.6%) |  | 9(47.4%) |  |
|  | AC (n=0) | / |  | / |  |
|  | BA (n=17) | 8(47.1%) |  | 11(64.7%) |  |
|  | BB (n=332) | 105(31.6%) |  | 158(47.6%) |  |
|  | BC (n=85) | 22(25.9%) |  | 34(40.0%) |  |
|  | CA (n=0) | / |  | / |  |
|  | CB (n=31) | 11(35.5%) |  | 16(51.6%) |  |
|  | CC (n=0) | / | 0.573 | / | 0.161 |
| 4 | AA (n=64) | 29(45.3%) |  | 39(60.9%) |  |
|  | AB (n=74) | 31(41.9%) |  | 44(59.5%) |  |
|  | AC (n=0) | / |  | / |  |
|  | BA (n=101) | 39(38.6%) |  | 51(50.5%) |  |
|  | BB (n=436) | 191(43.8%) |  | 258(59.2%) |  |
|  | BC (n=133) | 46(34.6%) |  | 64(48.1%) |  |
|  | CA (n=0) | / |  | / |  |
|  | CB (n=97) | 37(38.1%) |  | 58(59.8%) |  |
|  | CC (n=2) | 0 | 0.413 | 0 | 0.102 |
| 5 | AA (n=2) | 1(50.0%) |  | 1(50.0%) |  |
|  | AB (n=5) | 4(80.0%) |  | 4(80.0%) |  |
|  | AC (n=0) | / |  | / |  |
|  | BA (n=0) | / |  | / |  |
|  | BB (n=30) | 10(33.3%) |  | 12(40.0%) |  |
|  | BC (n=6) | 3(50.0%) |  | 4(66.7%) |  |
|  | CA (n=0) | / |  | / |  |
|  | CB (n=9) | 6(66.7%) |  | 7(77.8%) |  |
|  | CC (n=0) | / | 0.206 | / | 0.179 |
| 6 | AA (n=0) | / |  | / |  |
|  | AB (n=0) | / |  | / |  |
|  | AC (n=0) | / |  | / |  |
|  | BA (n=3) | 0 |  | 0 |  |
|  | BB (n=8) | 3 (37.5%) |  | 4 (50.0%) |  |
|  | BC (n=1) | 0 |  | 1(100.0%) |  |
|  | CA (n=0) | / |  | / |  |
|  | CB (n=3) | 1(33.3%) |  | 2(66.7%) |  |
|  | CC (n=0) | / | 0.815 | / | 0.354 |
